# Audit of physical health monitoring on admission to Mill Lodge (CAMHS Inpatient Unit, York)

**DOI:** 10.1192/bjo.2021.930

**Published:** 2021-06-18

**Authors:** Andreea Steiu, Emma Diggins, Nagulan Thevarajan

**Affiliations:** Mill Lodge, Leeds and York Partnership NHS Foundation Trust

## Abstract

**Aims:**

This audit aimed to evaluate the standard of initial physical health assessment that young people receive on admission to Mill Lodge.

Adherence to recommendation 2.6.3 of the service specification for Tier 4 CAMHS was assessed. Standard 2.6.3 of the service specification for Tier 4 child and adolescent mental health services states that “on admission all young people must have an initial assessment (including a risk assessment) and care-plan completed within 24 hours. Where admission is for day/in-patient care this will include a physical examination.” In line with this standard this audit will evaluate the use of physical examination, baseline blood tests and ECG carried out on young people.

**Background:**

Mental health problems in children and young people are associated with both short- and long-term physical health problems. It is therefore important that they undergo full physical health assessment on admission to a Tier 4 inpatient unit.

**Method:**

Electronic records were reviewed for all patients admitted within a 6 months period, between 1st August 2018 and 1st February 2019. Data were collected in March 2019 and entered directly into an excel spread sheet designed for data collection. A total of 23 patients were identified for inclusion in this audit.

Simple statistical analysis was carried out using excel.

**Result:**

Over 80% of patients who did not refuse had a completed physical examination (85%), blood results recorded (82%) and ECG (84%) within the first 24 hours of their admission. 100% of patients who did not refuse had bloods and ECG checked at some time during their admission, with 90% having a physical examination.

For several patients (3 physical examination, 2 bloods, 3 ECG), no reason was documented as to why the procedure or examination did not take place. For 1 patient, blood tests were delayed due to having no blood tubes available.

**Conclusion:**

Taken into account the result of this audit and bearing in mind the importance of physical examination as part of the admission process, it is important to try and support both regular Mill Lodge staff and on-call junior doctors to follow Standard 2.6.3's guidance around physical examination on admission to hospital. While good results were seen in many areas, the ward is not yet achieving the standard of 100%. A re-audit will take place in twelve months’ time to review recommendation and compliance.

